# Supplementary material for: MicroRNA-22 Is a Key Regulator of Lipid and Metabolic Homeostasis
Source: Int J Mol Sci. 2023 Aug 17;24(16):12870. doi: 10.3390/ijms241612870 (PMC10454516; doi:10.3390/ijms241612870)
Supplement: Supplementary file 1 [file ijms-24-12870-s001.zip › ijms-2480867-supplementary.pdf]

Fig.1 SUP

Panella et al.

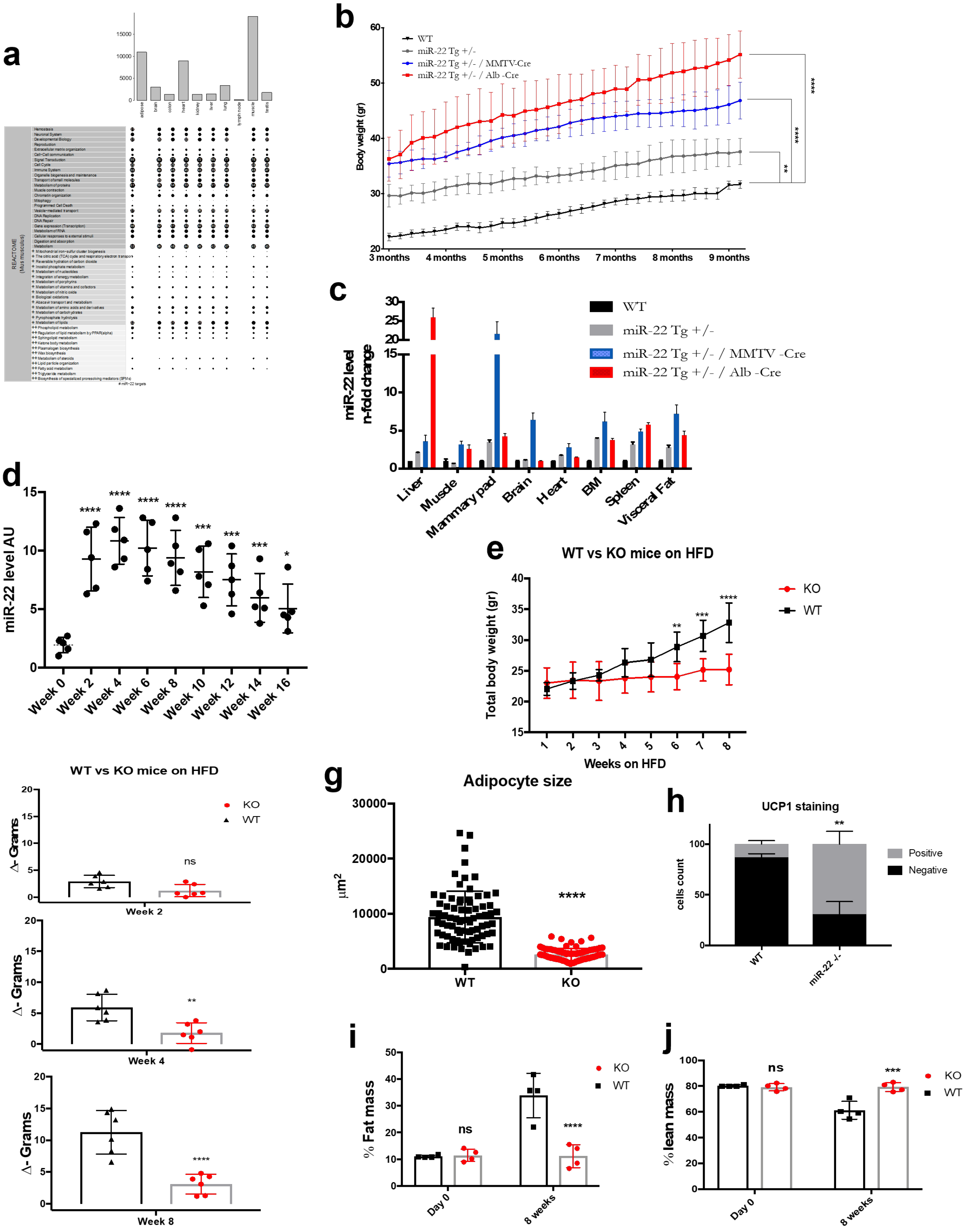

1.

Supplementary Figure 1 (related to Figure 1). Role of miR-22 in metabolism and obesity Bar plot showing miR-22 expression in mouse tissues (https://doi.org/10.1093/nar/gkw116), and many cell types. We used Illumina's Body Map v2 to identify which of the predicted miR-22 target genes are expressed (TPM > 1) in each of the ten tissues; tables show Reactome pathways in human and mice

b, Body weight comparison between WT and miR-22 transgenic mice without any Cre expression or expressing Cre under different promoters, fed on regular chow. The presence of miR-22 transgene alone is enough to induce weight gain in mice due to a leakiness in the C2 locus, however, when miR-22 level is induceed by Cre, the effect on body weight becomes emore pronounced and reaches the obesity threshold (>40g) (n=6 per cohort). **c**, miR-22 expression in different tissues of WT mice as well as miR-22 transgenic mice without Cre or expression of Cre under different promoters, showing that miR-22 levels are higher in miR-22 Tg mice, implying that the C2 locus in which the miR-22 transgene is cloned is leaky. Induction of Cre expression increases the overall level of miR-22 in almost all tissues compared to non–Cre mice with a strong induction in the Cre specific tissue (liver for Alb-Cre and mammary pad for MMTV-Cre). **d**, miR-22 levesl are upregulated in plasma of WT mice after 2 weeks on high fat diet (n=5 per cohort). **e,** Total body weight of WT and miR-22 deficient mice fed on HFD for 8 weeks (n=5 per cohort). **f,** Detailed total body weight of WT and miR-22 deficient mice fed on HFD for 8 weeks (n=5 per cohort) at week 2, week 4 and week 8. **g,** Quantification of adipocyte size in WAT of miR-22-KO and WT after 8 weeks on HFD. **h,** quantification of UCP-1 staining, related to Main Figure 2f; WAT from WT and miR-22-/- mice stained with an antibody against UCP-1 show a big difference in UCP-1 signal being stronger in the miR-22-/- sample compared to in WT. **i,** Echo MRI analysis of fat mass, expressed in percentage of total body weight, from WT and miR-22 deficient mice when fed with regular diet (day0) and after 8 weeks on HFD (8 weeks). The WT mice show an increase in fat mass, as expected, however miR-22$^{-/-}$ mice do not. **j,** Echo MRI analysis of lean mass, expressed in percentage of total body weight, present in WT and miR-22 deficient mice when fed on regular diet (day0) and after 8 weeks on HFD (8 weeks). WT mice show a decrease in lean mass not observed in miR-22$^{-/-}$ mice.

Fig.2 SUP **Panella et al.**

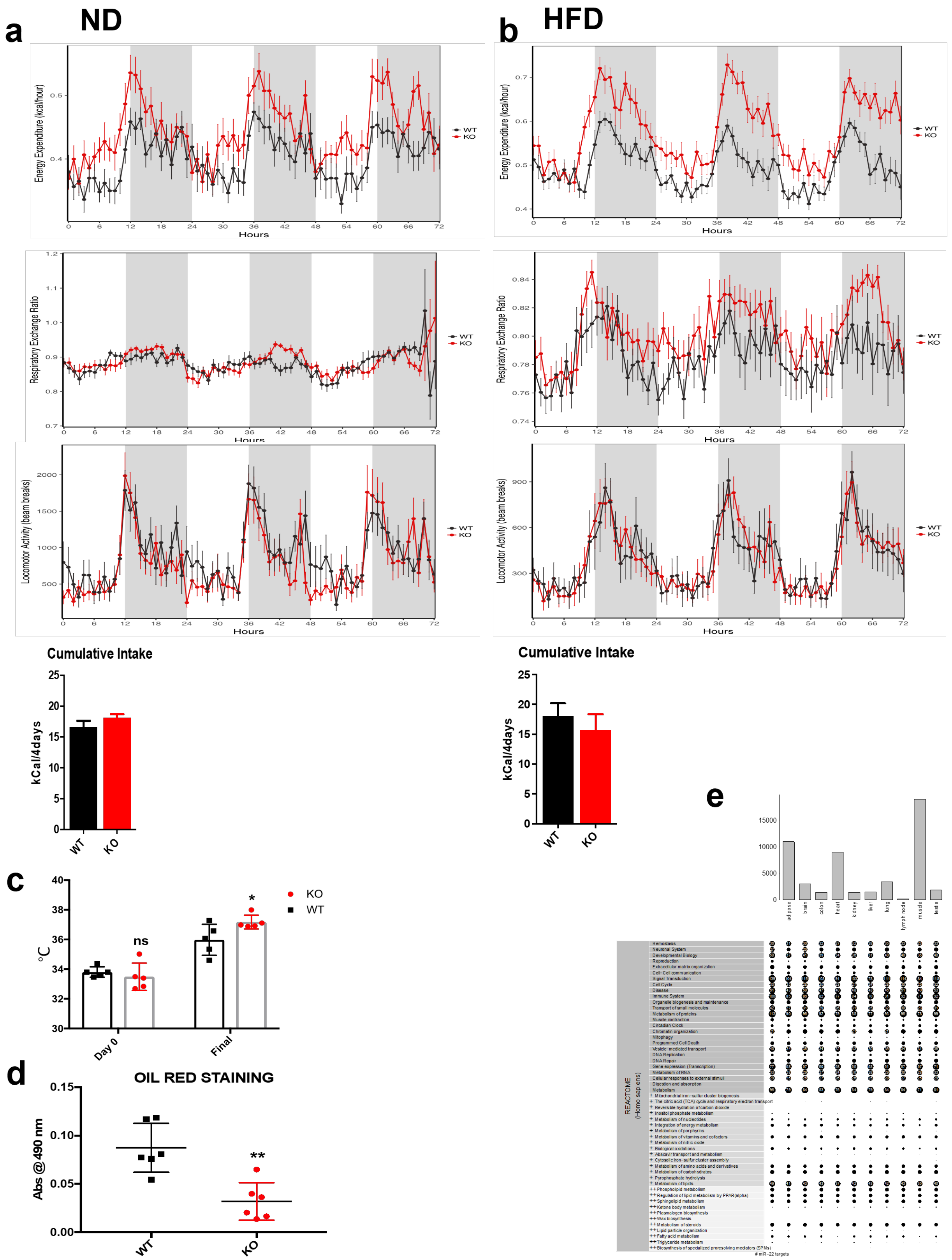

Supplementary Figure 2 (related to Figure 2). Genetic ablation of miR-22 prevents diet-induced obesity through metabolic rewiring. a, At baseline, WT and KO mice show no difference in energy expenditure, RER, locomotor activity and cumulative food intake. **b,** After 8W on HFD, KO mice have significantly higher energy expenditure than WT mice. There are no differences in RER, locomotor activity and cumulative food intake between WT and KO. **c,** quantification of thermal camera images, related to Main Figure 2e; at steady state WT and miR-22-/- mice show the same body temperature (n=5 per cohort), but after 8 weeks on HFD, KO mice exhibit

a statistically significant increase in body temperature. **d**, quantification of staining intensity between MEF cells from WT and miR-22-/- mice cultured in presence of adipose differentiation media for 8 days and then stained with Oil-Red-O. Staining of WT cells is much intense than miR-22 deficient cells, proving that miR-22 genetic inhibition cause and impairment in adipocyte differentiation, making miR-22 null cells less prone to differentiate in adipocytes. **e,** Bar plot of miR-22 expression in human tissues (https://doi.org/10.1093/nar/gkw116), showing that miR-22 is expressed in many cell types. We used Illuminas Body Map v2 to identify which of the predicted miR-22 target genes are expressed (TPM > 1) in each of the ten tissues; tables show Reactome pathways in human and mouse.

Waist-Hip Ratio: r=0.38, qvalue=0.0027
BMI: r=0.36, qvalue=0.0047
TRGs: r=0.34, qvalue=0.008
Alanine Aminotransferase: r=0.31, qvalue=0.015
ApoB: r=0.31, qvalue=0.015
Creatinine Clearance: r=0.3, qvalue=0.015
HDL cholesterol: r=-0.29, qvalue=0.016
HOMAIR: r=0.28, qvalue=0.017
Muscle Mass: r=-0.28, qvalue=0.017
sCRP: r=0.28, qvalue=0.017
OGTT Fasting Plasma Insulin: r=0.29, qvalue=0.017
Matsuda Insulin Sensitivity Index: r=-0.27, qvalue=0.019
HOMAIS: r=0.26, qvalue=0.022
OGTT 120' Plasma FFA: r=0.25, qvalue=0.025
Fat Mass: r=0.25, qvalue=0.028
Insulin AUC: r=0.21, qvalue=0.05
OGTT 30' Plasma Insulin: r=0.25. qvalue=0.028
Adiponectin: r=-0.24, qvalue=0.03
ApoA1: r=-0.22, qvalue=0.04

Table S1: Abdominal subcutaneous fat tissue miR-22 expression correlations with metabolic and anthropometric characteristics of METSIM study subjects (n=86). r: Spearman's rho. qvalue: Storey's qvalue.
BMI: Body Mass Index, TRGs: triglycerides, ApoB: Apolipoprotein B, HOMAIR: Homeostatic Model Assessment of Insulin Resistance, sCRP: serum C-reactive protein, OGTT: oral glucose tolerance test, HOMAIS: insulin secretion index based on Homeostatic Model Assessment, FFA: Free Fatty Acids, AUC: area under the curve, ApoA1: Apolipoprotein A1.
